# Extended Higher-Order Elements with Frequency-Doubled Parameters: The Hysteresis Loops Are Always of Type II

**DOI:** 10.3390/s23167179

**Published:** 2023-08-15

**Authors:** Zdeněk Biolek, Dalibor Biolek, Viera Biolková, Zdeněk Kolka

**Affiliations:** 1 Department of Electrical Engineering, University of Defence, 662 10 Brno, Czech Republic; zdenek.biolek@gmail.com; 2 Department of Microelectronics, Brno University of Technology, 616 00 Brno, Czech Republic; 3 Department of Radio Electronics, Brno University of Technology, 616 00 Brno, Czech Republic; biolkova@vut.cz (V.B.); kolka@vut.cz (Z.K.)

**Keywords:** higher-order element, extended element, generic element, pinched hysteresis loop

## Abstract

Current MEMS (Micro Electro Mechanical Systems) can be modeled by state-dependent elements that exhibit hysteretic behavior. Examples include capacitors and inductors whose capacitances and inductances are dependent on the instantaneous state of the electromechanical system, resistors whose resistances exhibit temperature changes when the elements are actually heated, etc. Regardless of the physical background, such hysteresis manifestations can be studied uniformly in the broader framework of generic and extended higher-order elements, in which a classification of hysteretic loops into types I and II is established. The loop type is an important dynamical parameter of an element, having the potential to indicate, for example, its (in)volatility. Thus far, there is no reliable criterion to determine the type of steady loop from the defining relations of an element. This work reports on one special class of extended elements that produces type II loops under all circumstances. The paper presents hitherto unpublished connections between the frequency-doubling parameters of an element and the type of its hysteresis loop. The new findings are expressed by several theorems that allow the type of hysteresis to be inferred from the frequency behavior of the element parameter or state, and vice versa. These procedures are demonstrated with examples and verified by computer simulations.

## 1. Introduction

Contemporary MEMS (Micro Electro Mechanical Systems), Micro/Nano Machines, microactuators, and sensors, along with the associated control, compensation, data, and signal processing systems [1,2], are often characterized by extremely complex behavior and nonlinear dynamics. In the interaction of subsystems whose behavior is subject to various physicochemical principles ranging from electrical engineering and mechanics to thermics, microfluidics, and, for example, biologic systems, phenomena often arise that deserve special attention. Their common denominators are specific memory effects. The first group of these effects is associated with memristor-type memory elements [3], the second with the fractional dynamics of some parts of MEMS [4].

Some of the specific manifestations of MEMS are enhanced by the extremely small size of these devices, thanks to which effects in nanoscale systems and complex materials begin to apply [5]. Evidently, the best known manifestation in this respect is the memory effect called memristance [6], observable in experimental data as typical hysteresis loops. These loops are observable in the electrical subsystems of MEMS in various coordinate systems, depending on the physical nature of the phenomenon. The corresponding subsystem is then referred to as memristive, memcapacitive, or meminductive [7]. Similar manifestations of hysteresis can also be provided by subsystems of non-electrical nature, especially the mechanical parts of MEMS, or they can be the results of interactions between subsystems of different nature. Such mechatronic mem-systems can be effectively studied using the generalization of so-called Chua’s table and predictive modeling methods [8] to the non-electrical domain [9], as well as the generalization of memristive, memcapacitive, and meminductive systems to so-called extended and generic Higher-Order Elements (HOEs) [10].

Various manifestations of hysteretic behavior can be observed in MEMS, ranging from the well-known voltage-displacement hysteresis patterns of piezoelectric actuators [11] to hysteresis loops pinched at the origin of the MEMS coordinate system (hereafter referred to as PHL-Pinched Hysteresis Loops), which exhibit memristive and similar effects. For example, the voltage-current PHLs can be observed in biomimetic memristors [12], UV sensors [13], or microtubular memristors [14]. Similar loops arise in the flux (time integral of voltage)-current coordinates in inductors fabricated by MEMS technology [15] and, in general, in systems governed by meminductive processes. Examples include inductive MEMS accelerometers [16], bimetallic cantilever microactuators [17], tactile sensors [18], or tunable RF MEMS inductors [19]. The memcapacitive processes then induce hysteresis in the charge (time integral of current)-voltage coordinates. This hysteresis has been reported in lipid bilayers, which have the potential to mimic cell membranes [20,21], or in electrostatic MEMS actuators [22]. It is shown in [22] that a bipolar comb drive as a quasi-static actuator of a MEMS-driven micromirror produces so-called narrow-band or selective hysteresis in the voltage-charge coordinates and that the corresponding hysteresis patterns can be substantially modified by DC biasing applied to the actuator.

The hysteresis in MEMS actuators and sensors is historically perceived as a negative phenomenon that deteriorates accuracy [23]. It can be a source of other problems, including instability [11]. From this perspective, the above hysteresis effects are seen as a natural manifestation of memristive and similar processes in a particular class of MEMS, which should either be tolerated or their effects attenuated by various compensation techniques [11,24]. On the other hand, however, there are applications whose essence lies in the direct exploitation of memristive and similar memory effects [25]. For example, the integration of memristors with MEMS is a promising way of improving the performance of collecting and processing data from sensors [26,27,28]. The contemporary literature describes many examples of the use of nano-memristive devices and nanowires for sensing or detecting temperature [29], gas [30], pH [31], cancer markers [32], DNA [33,34], UV-light [13], glucose [35], proteins [36], or, for example, for wearable non-contact breath sensing [37]. A very promising direction for the integration of sensors and memristors is in-sensor computing with memristors [38].

Fractional-order dynamics is another factor that can affect the behavior of MEMS. Although not the subject of this work, it is worth mentioning at least for completeness. The behavior of some systems or processes in MEMS is fundamentally governed by differential equations of non-integer order. Examples are diffusion processes and heat transfer through heterogeneous materials [39] or dynamic phenomena in mechanical viscoelastic materials [40]. Atypical behavior in the frequency domain (frequency characteristics with the steepness given by non-integer multiples of 20 db per decade) and in the time axis (transients will be governed by Mittag-Leffler functions [41] instead of exponential and damped harmonic functions) must be considered. Therefore, the designing and modeling of MEMS must be performed using fractional calculus. The relevant processes can be modeled by integrators of non-integral order, which are blocks with state vectors of infinite dimension [42]. As a consequence, it complicates the solution of the initial value problem in the fractional domain [43]. This has various practical consequences, for example, that the sensor response to a sensed quantity cannot simply be obtained as a sum of the forced response and the natural response to a finite number of initial conditions [44], as is common in integer-order linear systems. Another anomaly of MEMS of non-integer order is that the deviation of the actual system order from the classical integer order results in a modification of the damping of the system, which indirectly affects its stability. The issue is illustrated, for example, by publications analyzing transversely oscillating MEMS viscometers [45] or fractional-order arch MEMS resonators [46,47]. A number of papers focus on advanced control techniques that help to improve the stability and performance of fractional-order MEMS [45,46,47,48,49], which are implemented using the sliding mode or fractional-order controllers. Such units can then exhibit complex fractional-order dynamics.

In [50], inspired by the classical concept of accelerometers, the idea of fractional sensors is introduced, which in the future could be implemented by modern fabrication techniques similar to the way the CPE (Constant Phase Elements) is implemented in analog technology via approximating partition structures [51] or fractal geometry [52].

A bridge between the hysteretic behavior of MEMSs induced by internal mem-processes and MEMSs with fractional-order behavior is being created by attempts to redefine fractional-order memristors, memcapacitors, meminductors, and higher-order elements [53,54,55,56,57,58,59]. The corresponding fractional-order memory systems also exhibit hysteretic behavior, which is additionally modified by non-integer dynamics. Up until now, there have been no relevant results from this area that could be the basis for exploring these phenomena in MEMS.

This paper is concerned with the study of hysteresis effects in integer-order systems, which exhibit the memory effects discussed above and exhibited by various MEMS. It is shown in Section 2, which follows this Introduction, that the memristive, memcapacitive, and meminductive processes in contemporary MEMS can be modeled uniformly using the HOEs. It is recalled that pinched hysteresis loops can in principle be of two types (type I and type II), and examples of MEMS that produce these loops are given. The importance of studying these loops is explained. The concept of extended HOEs with frequency-doubled parameters is explained. In Section 3, the procedure for evaluating the type of hysteresis loop based on knowledge of the defining equation of the element modeling the MEMS is presented. Section 4 defines the concepts of the PIL (Parameter vs. Input Loop) and SIL (State vs. Input Loop) to characterize extended HOEs, and their connection with the already introduced concept of the PHL is explained. Section 5 is devoted to theorems that hold for extended elements with a frequency-doubled parameter. Section 6 formulates six theorems for the extended elements with frequency-doubled states. The application of the theorems is demonstrated by illustrative examples.

The main objective of this paper is to show that the extended HOEs for MEMS modeling, operating under sinusoidal excitation with frequency-doubled parameters, generate steady-state type II hysteresis loops without any exception. Thus, it is not simply a matter of native behavior; it is a matter of phenomena that occur inevitably, regardless of the initial conditions and parameters of the sinusoidal excitation. These hitherto unpublished new pieces of knowledge are summarized in the above-mentioned theorems.

## 2. MEMS Modeling via Extended HOEs: Type I and Type II Pinched Hysteresis Loops

The manifestations of memristance, memcapacitance, and meminductance are among the phenomena that have been widely studied recently in connection with the rapid development of MEMS and the technologies for new memory elements [60,61,62,63,64]. The common basis of these phenomena is the state-dependent parameter *P*(), which is the resistance, capacitance, or inductance. Thus, an electrical one-port is modeled whose parameter depends on the instantaneous state of the associated dynamical system, which can be both electrical and non-electrical in nature.

Memristive, memcapacitive, and meminductive one-ports are well established in world literature [6,7]. Today, inspired by the original notation of memristors [3,65], they are considered extended or generic memristors, memcapacitors, and meminductors [66]. These elements can be characterized by a unified form of the port and state equation:

(1) $$y=P\left(x,u\right)u,\;\dot{x}=f\left(x,u\right),$$

where *y* and *u* are the constitutive variables, ***x*** is a general state vector, and *P*() is a one-port parameter satisfying the condition:

(2) $$\underset{u\to 0}{\lim}P\left(x,u\right)u=0$$

for each state ***x***. The generic memelements have a parameter dependent only on the state ***x***, i.e., *P*() = *P*(***x***). The constitutive variables *u* and *y* for state-dependent resistors, capacitors, and inductors are given by the pairs of variables (*v*,*i*), (*v*,*q*), and (*φ*,*i*), with *q = i*^(−1)^ and *φ = v*^(−1)^ being charge and flux, respectively. The negative indices (−1) denote the first integrals with respect to time. Either an excitation role *u* or an output role *y* can be assigned to each of the quantities. It allows describing both the voltage and current representations of an element in a unified way given by Equations (1) and (2).

In general, the quantities *u* and *y* can be selected from a pair of quantities (*v*^(*α*)^,*i*^(*β*)^), where *v* and *i* are the voltage across the element and the current through the element, while the positive/negative integers *α* and *β* denote the order of differentiation/integration of the corresponding quantities with respect to time. The concept of constitutive quantities (*v*^(*α*)^,*i*^(*β*)^) appeared in [8] as part of the theory of ideal (*α*,*β*) elements, alias HOEs. These elements were introduced for the purpose of analyzing and modeling processes in the world of nanotechnology. An ideal HOE or ideal (*α*,*β*) element is a one-port defined by the algebraic constitutive relation *F*(*v*^(*α*)^,*i*^(*β*)^) = 0, where *F*() is in general a nonlinear function [8]. This concept allows treating the already known elements of resistor, capacitor, inductor, memristor, memcapacitor, and meminductor uniformly as HOEs (0,0), (0,−1), (−1,0), (−1,−1), (−1,−2), and (−2,−1). The element defined by (1) and (2), whose constitutive variables *u* and *y* can be the pairs (*v*^(*α*)^,*i*^(*β*)^), is called the Extended Higher-Order Element (Extended HOE) [10]. Generic elements differ from extended elements in the state-dependent parameter *P*(*x*), which does not depend on the excitation *u* [65].

In some real cases (see the example in Section 5.3), the port equation from the defining relation (1) may take a formally different form (see [6]) of the type:

(3) $$y=h\left(x,u\right),\;h\left(x,0\right)=0,$$

from which the *P*() parameter is derived by the procedure:

(4) $$P\left(x,u\right)=\left\{\begin{array}{cc} \frac{h\left(x,u\right)}{u} & \mathrm{for}\;u\neq 0\\ \underset{u\to 0}{\lim}\frac{h\left(x,u\right)}{u} & \mathrm{for}\;u=0 \end{array}\right..$$

One-ports (1) or (3) with intrinsic dynamics that modify their constitutive relations can be well used to model what happens in a variety of real-world processes across physical platforms [67,68]. Although some systems cannot be modeled with the exclusive use of one-ports arranged in suitable topologies [8], for many real-world complex systems, it can be carried out. Indeed, the variability of these one-ports is considerable since they are state-dependent elements with a general type of nonlinearity with respect to the excitation variable, where the state can be a vector of arbitrary physical quantities that respond to an external stimulus with any type of dynamics. In [69], it is shown how the memristive and memcapacitive effects, simultaneously acting in metal-oxide junctions, can be modeled in this way. Simpler examples that are typical for MEMS include memristive manifestations caused by the self-heating of an element [6], which causes a parameter change with the corresponding inertia. Another example, often observable in MEMS, is the memcapacitive phenomenon caused by capacitances dependent on external voltage or charge, be it through tunable geometry or material constants [5]. The same applies to meminductive systems, which are state-dependent inductances [5], where the dynamics of the state variable are controlled by an external current or flux. An example from mechanics is an innovative type of damper called the inerter [70], which is used, among others, to minimize the effect of external vibrations on the accuracy of measurements using micromechanical devices such as accelerometers, gyroscopes, or resonators. Its fluidic memory version [71], the so-called mem-dashpot, uses the internal helical channel to implement position-dependent inertia. In principle, it is a memcapacitor or meminductor, depending on the type of electro-mechanical analogy. It could be of interest in potential applications realizing micro-scale inertia in MEMS devices [72].

It follows from the dependence between the excitation quantity *u* and the output quantity *y* of the extended element (1) that a hysteresis arises due to the state-dependent parameter *P*(***x***,*u*). For bipolar excitation of *u* and under condition (2), this hysteresis is manifested by the loops in the *y − u* plane pinched at the origin. Pinched hysteresis loops (PHLs) belong to the fingerprints of extended and generic higher-order elements and thus also to their best known representatives: memristive, memcapacitive, and meminductive systems [66]. If the dynamics of an element respond to a periodic bipolar excitation *u* via a periodic steady state ***x*** whose frequency is an integer multiple of the excitation frequency, then, after the transient decays, the PHL has a steady form that is continuously redrawn during each successive excitation period.

As mentioned in [73], *…This particular signature (note: the PHL) has been explicitly observed in a number of devices for more than one century, while it can be extrapolated for devices that appeared as early as the dawn of the nineteenth century…* PHLs were first described in detail in [6] as unmistakable fingerprints of memristive systems, which are real objects of both natural and artificial nature. PHLs, as the results of repeated measurements on Pt-TiO2-Pt samples, led R. Williams’ team at Hewlett Packard Laboratories to report the first fabricated memristor, now known as the HP memristor [74]. The PHL features defined in the form of theorems in [6] for memristive systems are the same for PHLs of memcapacitive and meminductive systems, which has been verified many times by measurements on existing objects in which memcapacitive or meminductive processes play some role.

In 2011, two basic types of PHLs were recognized and named. In [5], *self-crossing* and *not-self-crossing* loops were described according to whether or not the loop changes its orientation after passing through the *y − u* origin. Another designation is the PHL of types I and II, according to whether the curve in the *P*(***x****,u*) *− u* plane forms one or two loops. In addition to the particular excitation, the loop type depends strongly on the nature of the physical processes that make up the dynamics of the element; it may also be affected by the type of nonlinearity of the *P*() parameter.

**Figure 1:**
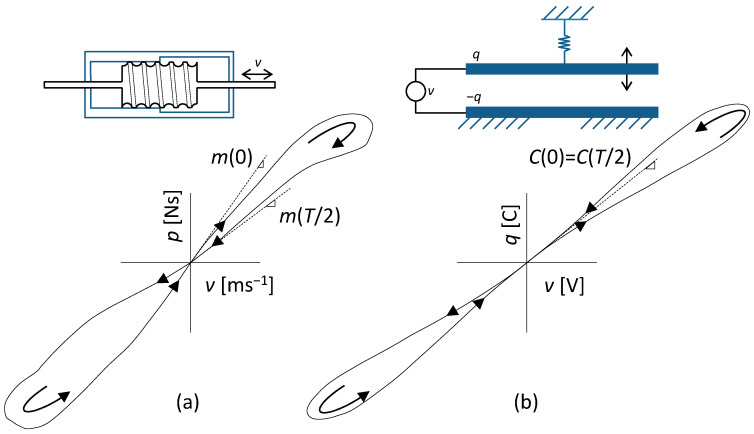
Demonstrations of PHLs of (**a**) fluid memdashpot (curve taken from [75]), type I; *p* and *v* are the moments of inertia and piston velocity; and (**b**) elastic memcapacitive system (curve taken from [5]), type II; *q* and *v* are the electric charge and voltage.

Figure 1 shows examples of type I and type II loops that can be obtained by measurements on existing devices under sinusoidal excitation *u*(*t*). Each loop consists of two lobes, which are plotted in the direction of the arrows in the first and third quadrants during the positive and negative half-periods of the sinusoidal excitation.

The loop in Figure 1a was obtained by measurements on a fluid inerter with position-dependent inertance [75] and is of type I. Since the two arms of the lobe pass through the origin at different angles, this means that the parameter *P*() of the element, which is the instantaneous mass of the moving part, has in both idle states a different value at the beginning and at the end of the excitation half-period. This type of memory behavior usually means that the element can be used as a non-volatile memory if other conditions are met [76].

An example of non-volatile elements that can be used in MEMS are so-called memsensors, or sensors and memory elements “in one”. A ZnO microrod device as a UV-light memsensor producing a type I hysteresis loop is presented in [13]. The concept of memsensors in terms of architecture and modeling is treated in [77]. The reading of the remembered state of a volatile memristive element is performed by a high-frequency signal *u*(*t*) of small amplitude. This exploits the fundamental regularity of the degradation of the hysteresis loop into a unique curve for high-frequency excitations [6]. This degradation is due to the fact that the oscillations of the state variable *x* vanish, so that, according to (1) or (3), the hysteresis also vanishes, and the high-frequency readout signal therefore cannot retroactively affect the memory state.

The type II loop in Figure 1b represents the steady-state response of an elastic memcapacitance system [5] to sinusoidal voltage excitation. It is an arrangement of two parallel plate electrodes, the lower one being fixed and the upper one elastically anchored via a spring. When a capacitor created in this way is charged from a voltage source, the plates are attracted by an electrostatic force depending on the charge supplied, thus changing the capacitance of the arrangement. It is a memcapacitance system described by Equation (1), where *u* is the voltage between the electrodes and *y* is the delivered charge. Since both arms of the loop lobe pass through the origin at the same angle, this means that the element loses memory when the excitation is disconnected.

Volatile memelements with type II hysteresis are commonly represented in MEMS in the form of inductors and capacitors dependent on the system geometry or temperature-dependent resistive elements with self-heating. In the vast majority of these cases, the element dynamics, represented by the state Equation (1) or (3), is set in motion by the square of the excitation quantity *u*(*t*), be it magnetic or electric field forces or thermal power [2]. For generic elements, this results in the element parameter oscillating at twice the frequency of the excitation quantity, and hence a centrally symmetric hysteresis loop, i.e., a type II loop, is inevitably formed.

## 3. Extended HOE: PHL Type Evaluation

Although the information about the loop type is contained in the very defining relation (1) of the element itself, it is not yet resolved how to determine this type from the knowledge of the excitation *u* and the functions *P*() and ***f***(). In this respect, the following partial results have been achieved so far:

According to [5], type II is often observed when *P*() and ***f***() are even functions of *u*. However, this is not a necessary condition for the occurrence of type II PHL. If ***f***() is an odd function of *u*, type I PHL is often observed.

The basic classification of PHLs was made [78] for ideal memristors. The initial classification into crossing-type, or type I, and non-crossing type, or type II, was completed by a number that expresses the order of contact between the two lobes of the loop. In this paper, an algorithm is derived to determine the loop type, and it is shown that for excitations that can be described as an odd function of time, ideal memristors always exhibit type I loops, and the odd-order touching is excluded.

The algorithm for evaluating the loop type is based on the assumption that the two arms of the PHL can be described in a certain neighborhood of the origin by the single-valued functions *y*^+^(*u*) and *y*^−^(*u*), where the indices + and *−* denote the loop arm along which the operating point travels from the first to the third and from the third to the first quadrant, respectively [78]. The algorithm evaluates the form of the function:

(5) $$\Delta \left(u\right)=y^{+}\left(u\right)-y^{-}\left(u\right),$$

where the superscripts + and − denote the positive and negative arms of the loop, respectively. If the function (5) has finite derivatives of all orders in a given neighborhood of the origin, then these derivatives at the point *u* = 0 contain information about the type of loop pinch. A crossing (CT) or non-crossing (NCT) loop is determined by whether or not the function (5) changes its sign during this crossing. The lowest order of the derivative that is responsible for this particular behavior is the order of touching. Let us use the symbols CT(*k*) and NCT(*k*) to denote crossing and non-crossing passes, respectively; *k* is the order of touching of the arms.

The loop type and order of touching *k* are determined by evaluating the sequence:

(6) $$\left\{\Delta_{i}\right\}=\underset{u\to 0}{\lim}\left\{\frac{d^{i}\Delta \left(u\right)}{du^{i}}\right\}$$

for *i* = 1, 2, …. The order of touching *k* is the index of the last zero term in the sequence (6). It is shown in [78] that an even *k* always denotes the CT type and an odd *k* the NCT type.

The terms of the sequence (6) can be written according to [78] in the form:

(7) $$\Delta_{i}=i\underset{u\to 0}{\lim}{\left[\frac{d^{i-1}P}{du^{i-1}}\right]}_{-}^{+}=i\left(P_{+}^{\left(i-1\right)}-P_{-}^{\left(i-1\right)}\right),$$

where the indices along the square brackets mean that the content of the brackets for the negative arm of the loop is subtracted from the content of the brackets for the positive arm of the loop. By comparing (6) and (7), it can be concluded that the order of touching *k* is also the lowest order of the derivative of the parameter *P* with respect to *u*, for which:

(8) $$P_{+}^{\left(k\right)}\neq P_{-}^{\left(k\right)}$$

In [79], a classification of PHLs for memristive systems according to the type of steady-state response to sinusoidal excitation is introduced. If the state variable ***x***(*t*) oscillates at a frequency that is an *odd* multiple of the excitation frequency, then the native loop type is type I, i.e., the crossing type. The native type implies a natural behavior that always occurs except in precisely described situations. Similarly, if the state variable ***x***(*t*) oscillates at a frequency that is an *even* multiple of the excitation frequency, then the native loop type is type II, i.e., non-crossing type. In [10], these findings were generalized to extended HOEs.

“Frequency doubled parameters” are encountered in physical systems where the force action depends on the square of the excitation quantity. In MEMS based on capacitive effects, for example, this is manifested by the capacitance oscillating at twice the frequency of the voltage excitation [80]. This is a logical consequence of the periodic changes in geometry in the rhythm of electrostatic forces, which are proportionate to the square of the applied voltage. Examples can be found in a variety of applications across physical disciplines, such as in biomimetics in the study of memcapacitance effects in synthetic cell membranes [20]. Analogous phenomena are observed in MEMS exploiting state changes in inductance, which result from the quadratic dependence of the magnetic force on the excitation current [81,82]. On the other hand, in memristive systems based on thermal heating due to flowing current, the dynamics are determined by thermal power, i.e., by the quadratic dependence of voltage or current excitation. This principle is applied, for example, in thermistors [6,83], in memristive ion channel-doped biomembranes [12], or in Mott memristors [84]. In all these cases, we observe type II PHLs in the steady state.

Using an example of the memristive system, it is shown in [85] that a slight distortion of pure sinusoidal excitation can lead to a change of PHL from type II to type I. Thus, it only makes sense to consider the PHL type in conjunction with a particular type of excitation. For the purposes of this paper, we will assume excitation exclusively in the form of a sinusoidal signal.

(9) $$u\left(t\right)=U\sin\left(\omega t\right)$$

where *U* is the amplitude and *ω* is the angular frequency.

## 4. Characteristics of Extended HOE: PHL, PIL, and SIL

The dynamics of nonlinear systems subjected to periodic excitation are often evaluated using Lissajous patterns, which are plotted as parametric curves between the excitation quantity and the corresponding response. If the response is an easily measurable or computable quantity, the measurement can be simply implemented using an oscilloscope. Different types of these patterns tend to be included in publications reporting the development of new memory elements [5,12,79,80,86]. In addition to the *y* − *u* PHLs, these are most often Lissajous patterns presenting the dynamics of the element parameters themselves (e.g., biomembrane capacitance) or physical quantities representing the element state (e.g., membrane area and thickness). For the last two types of curves, we now introduce abbreviations in order to make the paper clearer.

The dynamics of the extended element (1) can be studied using the dependence of the parameter *P* of the element on the excitation *u*. In this paper, we will denote it as the “Parameter vs. Input Loop” (PIL) according to the loop it represents in the *P − u* plane. Depending on whether it is a simple closed curve or a loop divided by a crossing into two separate lobes, a classification of PHLs in the *y − u* plane into types I or II has been developed [5].

The PIL is the result of a nonlinear transformation of the Lissayouss pattern formed via steady waveforms of the quantities ***x*** and *u* in the *P − u* plane. The *x_i_ − u* Lissayous pattern exists for each component *x_i_* of the vector ***x***. Each of these patterns will be called “State vs. Input Loop” (SIL).

**Figure 2:**
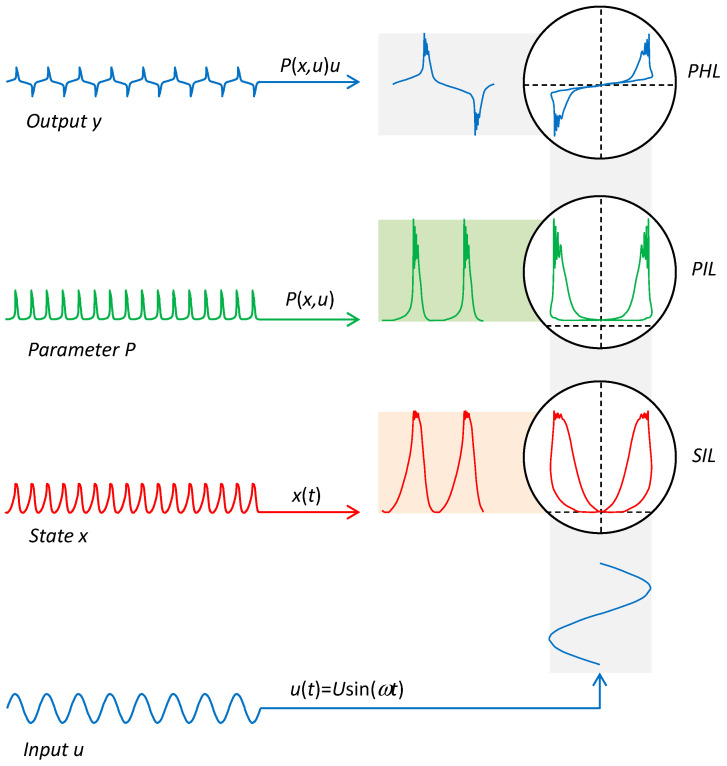
Relationships between the State vs. Input Loop (SIL), Parameter vs. Input Loop (PIL), and Pinched Hysteresis Loop (PHL) of the extended element.

The relationships between the SIL, PIL, and PHL are shown in Figure 2. The waveforms shown correspond to the waveforms of charge (*y*), capacitance (*P*), mechanical deflection (*x*), and voltage (*u*) in the application of memcapacitive MEMS from [80].

Generally, the PIL pattern of the extended element need not be axially symmetric, just as the PHL need not be a centrally symmetric curve. However, certain rules apply to elements with frequency-doubled parameters or frequency-doubled states, which will be discussed in the next two sections.

## 5. Extended Higher-Order Element with Frequency-Doubled Parameter

Consider an extended element (1) excited by a sinusoidal signal (9) of frequency *ω*. Let the parameter *P* of the element respond to this excitation with a periodic steady waveform of frequency 2*ω*. Then the following two theorems hold:

### 5.1. PIL Features

**Theorem** **1.** *The steady trajectory of the extended HOE with frequency-doubled parameter forms in the P − u plane the crossing-type PIL, which is axially symmetric around the line u = 0.*

**Proof.**  One whole period of the waveform *P* is repeated in the course of each half-period of sinusoidal excitation *u*. Therefore, the lobes drawn in the right and left half-planes of the *P − u* plane during the first and second half-periods of sinusoidal excitation must be axially symmetric with respect to each other along the axis of symmetry *u* = 0. The connection between the lobes is represented by a point lying on the axis of symmetry at *P*_+_ = *P_−_*. Since each half-period of excitation *u* is a copy of the previous one but with the opposite sign, the lobes have opposite orientations to each other in terms of the motion of the working point and thus form a crossing-type PIL. □

### 5.2. PHL Features

**Theorem** **2.** *The pinched hysteresis loop of the extended HOE with frequency-doubled parameter is centrally symmetric around the origin, and the orientation is always of type II, i.e., the noncrossing type.*

**Proof.**  Geometrically, a PHL lobe located in the left half-plane of the *y − u* plane is formed from a lobe located in its right half-plane by composing two axial symmetries: ① around the *u* = 0 axis (PIL axial symmetry); ② around the *y* = 0 axis (multiplying by the negative half-wave of the driving sinusoid). The composition of the two symmetries around mutually perpendicular axes is equivalent to the central symmetry around the intersection of the two axes. The result will be a PHL centrally symmetric around the origin of the coordinates.

Axial symmetry changes the orientation of the curve. A composition of two axial symmetries leading from one lobe of the PHL to the other will preserve the orientation of the original lobe. The PHL will be of type II. □

**Figure 3:**
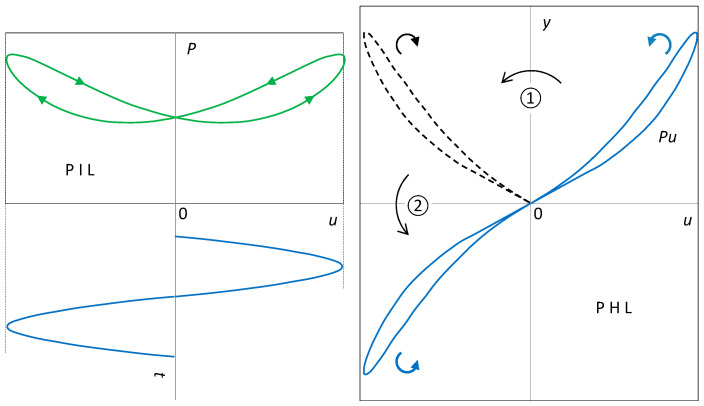
PIL-to-PHL transformation.

The composition of the two axial symmetries is shown in Figure 3. The right lobe of the PHL is formed from the PIL by the operation *y* = *Pu*, where *u* is the positive half-wave of the excitation. As a result, the orientation of the lobe is preserved. The axial symmetry around the *y* axis leads to a lobe that must be subjected to a second axial symmetry, this time around the *u* axis. This second transformation corresponds to the change in the half-wave of excitation from positive to negative. The first transformation reverses the orientation of the lobe; the second brings it back. The PHL is therefore of type II.

It follows from the above that the crossing-type PIL inevitably implies the non-crossing-type PHL, and vice versa.

Both theorems have simple mathematical expressions. For the derivatives of various orders of the parameter *P* with respect to the excitation *u* at the instants of zero crossing, the following must hold:

(10) $$P_{+}^{\left(1\right)}=-P_{-}^{\left(1\right)},P_{+}^{\left(2\right)}=P_{-}^{\left(2\right)},..,P_{+}^{\left(i-1\right)}={\left(-1\right)}^{i-1}P_{-}^{\left(i-1\right)}$$

where the indices + and *−* denote the correspondence between the positive and negative arms of the PHL. It follows from (10) that for the terms (7) of sequence (6) with indices *i* > 1, it holds:

(11) $$\Delta_{i}=\left\{\begin{array}{cc} 0 & \mathrm{for}\;i=\mathrm{odd}\\ 2iP_{+}^{\left(i-1\right)} & \mathrm{for}\;i=\mathrm{even} \end{array}\right..$$

All odd terms in the sequence are zero, which means that the PHL cannot be of type I. The odd index *k* of the last zero term denotes the order of touching of two arms of the PHL of the NCT(*k*) type.

### 5.3. Illustrative Example: The Graetz Bridge with Inductive Load

**Figure 4:**
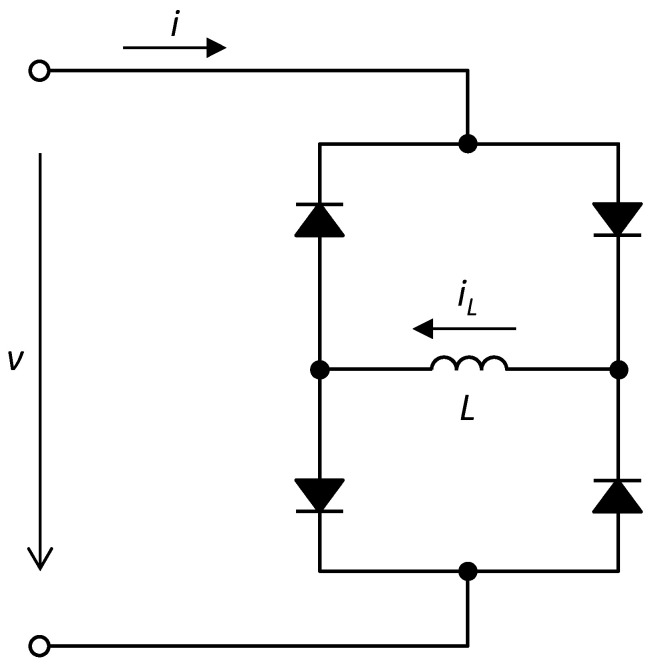
The Graetz rectifier with an inductive load as an extended memristor.

It was published for the first time in [87] that the Graetz rectifier with an LCR-type reactive load behaves like an extended memristor with respect to a voltage source. Figure 4 shows a modification of the original circuit consisting of the use of a purely inductive load [88]. The silicon diodes are of type 1N4148, and the inductance *L* has a value of 100 mH.

According to [88], the port equation of the extended element is:

(12) $$i=\left(i_{L}+2I_{s}\right)\tanh\left(\rho v\right)$$

where the inductor current *i_L_* serves as a state variable, *ρ* = (*2nV_T_)^−^*^1^, and the diode parameters *n*, *V_T_*, and *I_s_* are the emission coefficient, temperature voltage, and saturation current, respectively. According to [88], the state equation has the form:

(13) $$\frac{di_{L}}{dt}=\frac{1}{\rho L}\ln\left(\frac{2I_{s}\cosh\left(\rho v\right)}{i_{L}+2I_{s}}\right)$$

The nonlinear parameter of the extended memristor is its memristance *R_M_.* According to (4), it is defined from the port Equation (12) as the reciprocal of the memductance *G_M_*:

(14) $$G_{M}\left(i_{L},v\right)=\left\{\begin{array}{cc} \left(i_{L}+2I_{s}\right)\frac{\tanh\left(\rho v\right)}{v} & \mathrm{for}\;v\neq 0\\ \left(i_{L}+2I_{s}\right)\rho & \mathrm{for}\;v=0 \end{array}\right.$$

**Figure 5:**
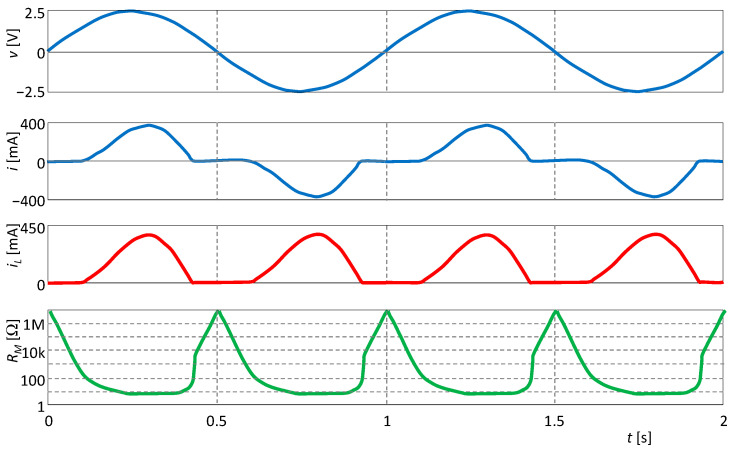
Waveforms of excitation voltage *v*, current response *i*, state variable *i_L_*, and memristance *R_M_ = v/i*.

The circuit is excited by a sine-wave voltage with an amplitude of 2.5 V and a frequency of 1 Hz. In Figure 5, the waveforms of the individual quantities are presented, showing that the parameter, which is the memristance *R_M_*, oscillates at twice the excitation frequency. Thus, the PHL and PIL must follow Theorems 1 and 2.

**Figure 6:**
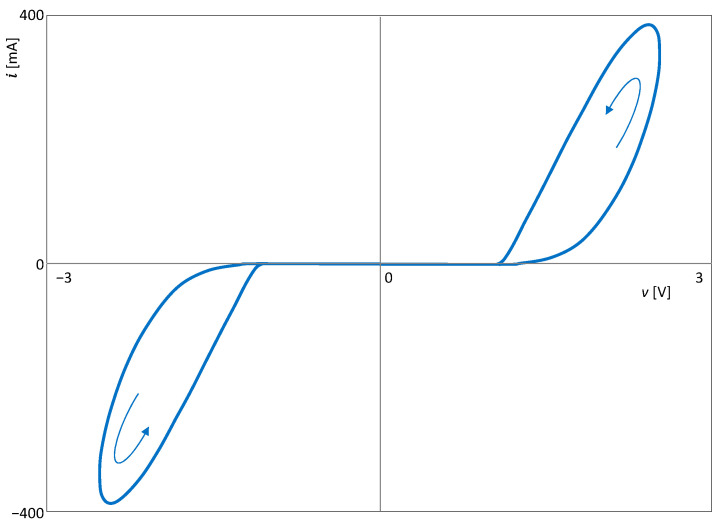
Non-crossing-type hysteresis loop of the extended memristor from Figure 4.

The type II hysteresis loop is drawn in the *v − i* plane, as demonstrated in Figure 6. This result is consistent with Theorem 2.

**Figure 7:**
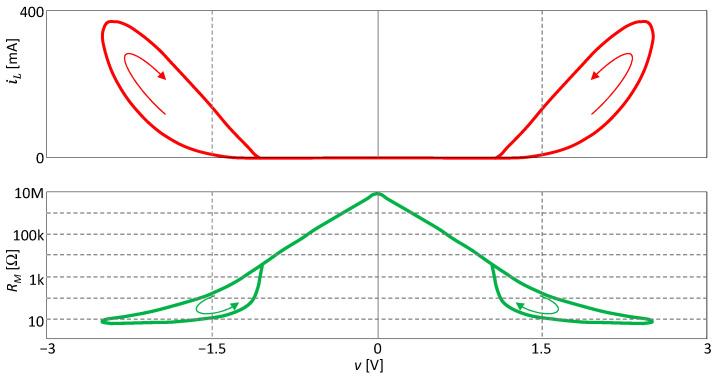
Crossing-type loops of SIL and PIL of the extended memristor from Figure 4.

Figure 7 shows the SIL and PIL curves. Both are of the crossing type. In accordance with Theorem 1, the PIL is symmetric around the axis *v* = 0.

For the particular characteristic (12), the first two terms of the sequence (6) have the following form:

(15) $$\Delta_{1}={\left[i_{L}+2I_{s}\right]}_{-}^{+}=0,\;\Delta_{2}=2\rho {\left[\frac{di_{L}}{dv}\right]}_{-}^{+}\neq 0$$

Thus, the index of the last zero member of the sequence is 1, which is also the order of touching of the PHL arms at the origin. The loop in Figure 6 is therefore of the NCT(1) type, although it consists of two distinctly isolated lobes. The cause of this phenomenon is the behavior of the state variable *i_L_*, which does not respond to changes in the voltage excitation for a long time, as shown by the SIL curve in Figure 7. The term Δ_2_, which affects the order of the touching of the arms, therefore has a very small value. Further analysis shows that all subsequent terms with even indices will also have negligible values since their magnitude will be proportional to the steepness of the *i*-th order of the SIL curve for *v =* 0. The two arms only become significantly “unlinked” when terms significantly different from zero begin to appear in the sequence {Δ*_i_*}. Hence, it seems from the extreme isolation of the two lobes that the order of touching is much higher than 1. For higher excitation frequencies, this effect is not as pronounced, and the PHL shape changes compared to Figure 6. However, the type II central symmetry is preserved, as shown in the original paper [88].

## 6. Extended Higher-Order Element with Frequency-Doubled State

Let us assume that the extended element (1) responds to the sinusoidal excitation (9) by a state vector, which in the steady state has a periodic waveform, the frequency of which is twice the frequency of the excitation. The following theorems hold for such an element.

### 6.1. PHL and PIL Features in the Case of a Generic Element

**Theorem** **3.** *The generic element with a frequency-doubled steady state produces an axial-symmetric PIL with odd symmetry, and its PHL is centrally symmetric and of non-crossing type, thus type II loop.*

**Proof.**  If the steady-state vector ***x*** is a periodic function of time whose frequency is twice the frequency of the excitation, then the waveforms of the function *P*(***x***) within the first and second half-periods of excitation are identical. The axial-symmetric PIL with an odd symmetry and the even-symmetric PHL are then direct consequences of Theorems 1 and 2. □

### 6.2. PHL Type in the Case of an Extended Element

**Theorem** **4.** *An extended element with a frequency-doubled state cannot produce the PHL of CT(0) type, i.e., crossing-type, without touching the arms.*

**Proof.**  If the steady-state vector ***x*** is a periodic function of time with twice the frequency of the excitation frequency, then:

(16) $$P_{+}=P_{-}$$

i.e., the parameter *P* will take the same value every time the excitation passes through the zero level. Thus, the PHL arms must have a touching order of 1 or higher. □

**Theorem** **5.** *An extended element with a frequency-doubled state can produce the PHL of type I or II depending on the type of nonlinearity of parameter P with respect to excitation u.*

**Proof.**  We prove the claim on an extended element with an associated dynamical system with scalar state *x*. The derivative of the parameter *P* with respect to excitation *u* is:

(17) $$P^{\left(1\right)}=\frac{dP}{du}=\frac{\partial P}{\partial x}\frac{dx}{du}+\frac{\partial P}{\partial u}.$$

The second derivative is:

(18) $$P^{\left(2\right)}=\frac{d^{2}P}{du^{2}}=\frac{\partial^{2}P}{\partial x^{2}}{\left(\frac{dx}{du}\right)}^{2}+\frac{\partial P}{\partial x}\frac{d^{2}x}{du^{2}}+2\frac{\partial^{2}P}{\partial x\partial u}\frac{dx}{du}+\frac{\partial^{2}P}{\partial u^{2}}.$$

An analysis of *P*^(1)^ shows that d*x*/d*u* changes its sign with each pass of the excitation through zero, but its absolute value remains the same. Since all the other terms of (17) maintain their values during these passes, Δ_2_ ≠ 0 according to (7). Then, according to (8), the PHL is of the NCT(1) type. However, this conclusion does not hold for special forms of the function *P*(*x*,*u*) that satisfy the condition:

(19) $${\left.\frac{\partial P}{\partial x}\right|}_{u=0}=0$$

Then Δ_2_ = 0, which excludes the NCT(1) type. Thus, the type of PHL could be decided by the relation (18) for *P*^(2)^. Similar reasoning over *P*^(2)^ leads to the conclusion that Δ_3_ ≠ 0. Thus, for the general form of the function *P*, the loop is of the CT(2) type. An exception would be special forms of the function *P*(*x*,*u*) satisfying the condition:

(20) $${\left.\frac{\partial^{2}P}{\partial x\partial u}\right|}_{u=0}=0.$$

Then Δ_3_ =0, which rules out the CT(2) type. To determine the loop type, one would need to investigate the relation for the next higher-order derivative of the parameter, i.e., *P*^(3)^.

The native PHL of the extended element with frequency-doubled state is therefore of the NCT(1) type, but what the actual type is depends on the specific form of the function *P*(***x***,*u*). □

An example of a specific form of the function *P* is as follows:

(21) $$P\left(x,u\right)=P_{0}+F\left(x\right)G\left(u\right),$$

where *P*_0_ is a constant, *F*() and *G*() are infinitely differentiable functions, and *G*(0) = 0. The element with this type of nonlinearity satisfies the condition (19), and if it provides frequency-doubled state dynamics, it cannot produce PHLs of the NCT(1) type. For different forms of the function *G*(*u*), the PHL type can vary between CT and NCT, and the order of touching can vary too.

**Theorem** **6.** *An extended element under sinusoidal excitation with a frequency-doubled state whose parameter P(**x**,u) is an even function with respect to the variable u produces PHL of type II.*

**Proof.**  Given the assumptions, the following applies:

(22) $$P\left(x\left(t+T/2\right),u\left(t+T/2\right)\right)=P\left(x\left(t\right),-u\left(t\right)\right)=P\left(x\left(t\right),u\left(t\right)\right),$$

i.e., the waveform of the parameter *P* has half the period and therefore twice the frequency compared to the excitation *u*. According to Theorem 2, the PHL must be of type II. □

The evenness condition of the function *P*() with respect to the variable *u* is a sufficient condition, not a necessary one. Section 6.3 will give an example of an element with a frequency-doubled state whose parameter is not an even function with respect to the excitation and still oscillates at twice the excitation frequency.

An example of an element with a parameter that is an even function with respect to the excitation variable *u* is the Graetz circuit presented in Section 5.3.

### 6.3. Illustrative Example: Extended Memristor

Let us demonstrate the validity of Theorems 5 and 6 with the example of an extended element defined by the relations:

(23) $$y=\underset{P\left(x,u\right)}{\underset{︸}{\left(P_{0}+P_{1}xu^{m}\right)}}u,\;\dot{x}=k_{1}u^{n}-k_{2}x,$$

where *P*_0_*, P*_1_*, k*_1_*, k*_2_*, m,* and *n* are constants, the last two being positive integers. The parameter *P*(*x*,*u*) is of the form (21).

For different combinations of constants, the general model (23) is able to describe a variety of applications of MEMS, including smart sensors and biomimetic systems. For generic frequency-doubled state elements, the typical case is *m* = 0, *n* = 2. According to Theorem 3, their PHL will always be of type II. For example, a synthetic membrane, formed by the contact of two lipid droplets, behaves as a generic memcapacitor due to the cooperation of the physical processes Electrowetting (EW) and Electrocompression (EC) [20]. If the quantities *y* and *u* are the voltage and charge and the state variable *x* is the membrane area, then the EW process is governed by the model (23). The same model governs a bistable, non-volatile MEMS membrane exhibiting chaotic behavior [80] when the components of the state vector are the position and velocity of the membrane. The same model describes the voltage and current dynamics of a temperature-dependent resistor [85], with a linear temperature dependence of the resistivity. In all cases, the experiments described in [20,80,85] show type II hysteresis. This is in agreement with Theorem 3.

The model (23) is also suitable for elements operating in the non-volatile mode, such as ideal generic memristors, memcapacitors, or meminductors. The combination of constants in these cases will be *m* = 0, *n* = 1, *k*_1_ = 1, and *k*_2_ = 0. It is well known that these elements produce type I hysteresis loops during sinusoidal excitation [76].

The behavior of the element (23) will be verified by computer simulation. Without loss of generality, this element can be considered an extended memristor, where *y* is the voltage and *u* is the current. Let the amplitude and frequency of the current sinusoidal excitation (10) be *I =* 0.1 A and *f* = *ω*/(2*π*) = 10 Hz. In the three subsequent simulations, the constants *P*_0_ =20 and *k*_2_ =100 remain fixed. The other constants of the quartet *{P*_1_, *k*_1_, *m*, *n*} will take the values {2000, 10, 1, 2}, {20,000, 10, 2, 2}, and {2000, 1, 1, 1} in simulations ❶, ❷, and ❸, respectively.

**Figure 8:**
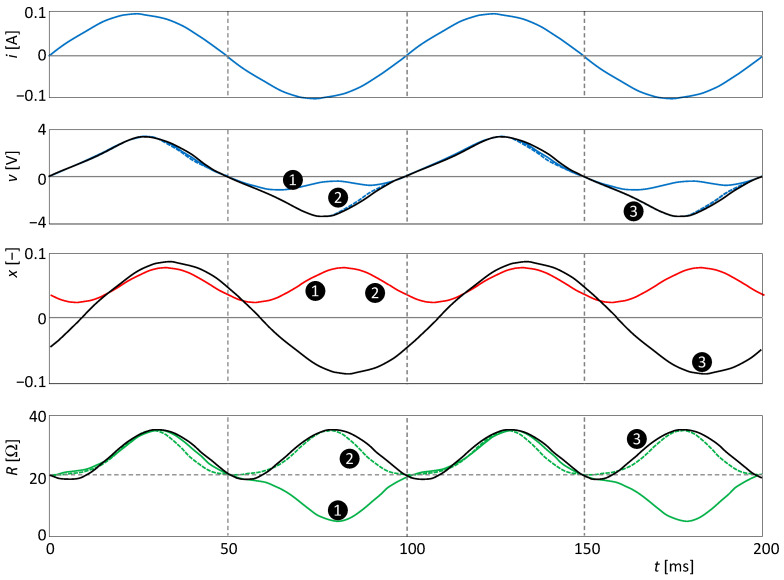
Waveforms of the excitation current *i*, voltage response *v*, state variable *x*, and resistance *R*.

The waveforms of input, output, state variables, and steady-state resistance are presented in Figure 8.

Since *n =* 2 in the cases ❶ and ❷, the state variable will oscillate at twice the excitation frequency.

Since *n* = 1 for the simulation ❸, the frequency of state *x* is equal to the frequency of current excitation *i*. In this case, the doubling of the frequency of the waveform of the resistance is worth noting. This doubling arises from the nonlinearity of the parameter (23) by combining the sinusoidal excitation signals *i = Isin*(*ωt*) and the steady state variable *x = Xsin*(*ωt* + *φ*), where *X* is the amplitude of the sinusoidal steady state response and *φ* is its phase delay with respect to the excitation signal. The resistance parameter *P≡R* from Figure 8 is governed by the relation:

(24) $$P=P_{0}+P_{1}XI\sin\left(\omega t\right)\sin\left(\omega t+\varphi \right)=\frac{P_{0}+P_{1}XI}{2}\left[\cos\varphi +\cos\left(2\omega t+\varphi \right)\right],$$

i.e., it has a nonzero DC component and a zero fundamental harmonic component. Thus, the parameter can oscillate at twice the frequency of the excitation without being an even function of that excitation.

**Figure 9:**
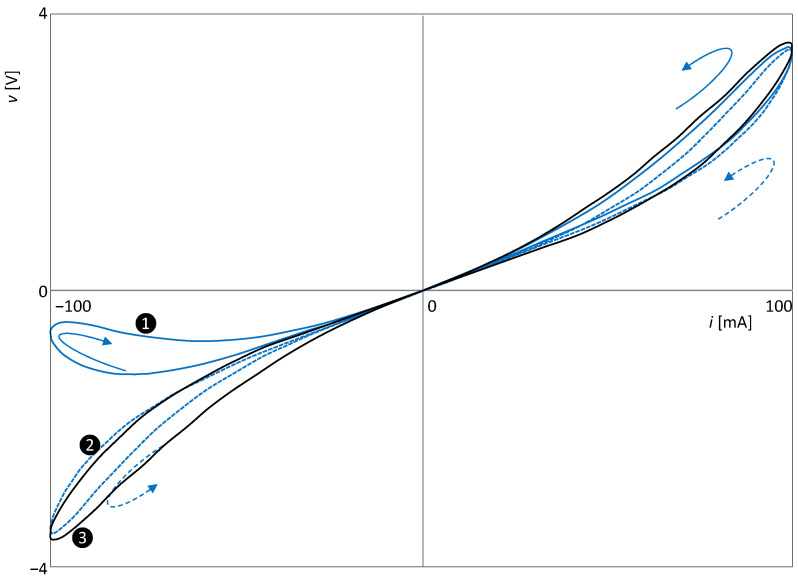
PHLs of the extended memristor according to (24): ❶ is nonsymmetric PHL of type I, ❷ and ❸ are symmetric PHLs of type II.

According to Theorem 2, the PHLs in the cases ❷ and ❸ will be of type II since it is an element with a frequency-doubled parameter. The case ❷ is also consistent with Theorem 6, since for *m =* 2, the resistance is an even function of the excitation current *i*. Figure 9 plots the steady forms of the PHLs for all three simulation runs.

For simulation ❶, *m =* 1, and therefore the condition (19) is satisfied but not the condition (20). Therefore, the element in steady state will produce a CT(2)-type PHL.

A similar consideration of the conditions of simulation ❷ leads to the conclusion that the PHL will be of the NCT(3) type.

Simulation ❸ leads to the PHL of the NCT(1) type.

**Figure 10:**
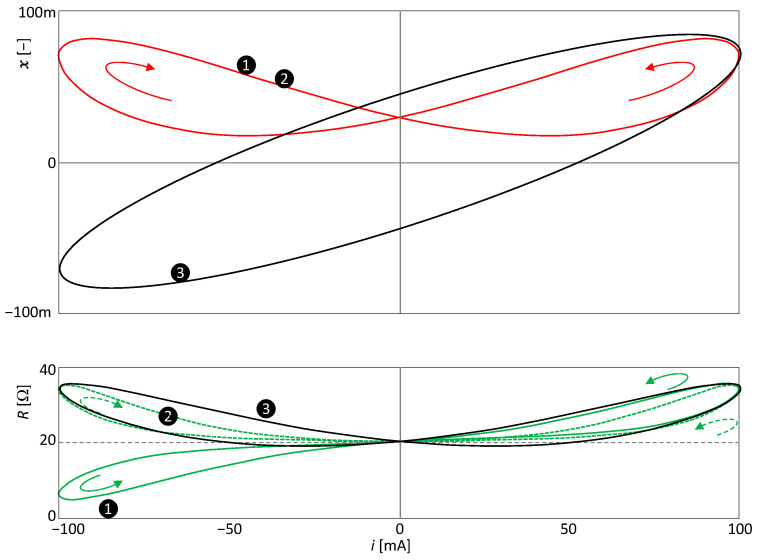
SIL and PIL of the extended memristor, according to (23). A solid line/dashed line indicates a non-crossing/crossing-type PIL.

Figure 10 shows the waveforms of the corresponding SIL and PIL curves. The SIL curve is not affected by the change in the exponent *m*. Comparison of Figure 9 and Figure 10 further confirms the regularity that the type of crossing of the PIL is always opposite the type of crossing of the PHL.

It is worth noting that although in the case of ❶ the resistance oscillates at the same frequency as the excitation frequency and therefore the element has the type I PHL, the PIL forms two separate lobes. Note that the original classification of PHLs into types I and II has been introduced based on whether the PIL is formed by one continuous curve or a curve divided into two lobes [5].

## 7. Implications of New Theorems

The theorems published in this paper have a number of practical consequences. For example, the following conclusions can be drawn from a mere glance at the steady form of the PHL or PIL:

- If the steady PHL is of type I or is centrally asymmetric regardless of the loop type, then it is not an element with a frequency-doubled parameter (implied by Theorem 2).
- If the generic element produces a steady PHL of type I or the PHL is centrally asymmetric regardless of the loop type, then it is not an element with a frequency-doubled state (implied by Theorem 3).
- If an element generates a PHL of the classical CT(0) type, it means that at least one of its state variables does not oscillate at twice the frequency of the excitation signal (following from Theorem 4).

On the basis of the theorems published in this paper, it is possible to correct some previously conceived ideas:

- If the Parameter vs. Input Loop (PIL) consists of two lobes, this does not mean that a type II PHL will be generated (see the illustrative example in Section 6.3). Thus, the current classification of loops into types I and II based on the number of PIL loops [5] does not always correspond to reality.
- If the state variable oscillates at twice the frequency of the excitation variable, it does not follow that the PHL must be of type II. However, this always follows for generic elements.
- If the state variable oscillates at the same frequency as the excitation variable, it does not mean that the PHL must be of type I.

## 8. Conclusions

Contemporary MEMS are complex nonlinear systems where state-dependent elements of different physical natures are interconnected. These elements can, in many cases, be modeled as extended higher-order elements. The results of this work enrich the theoretical basis for analyzing and modeling hysteresis, which is a common phenomenon in such systems.

Extended elements with frequency-doubled parameters form an interesting and widely used subset of extended higher-order elements. In the steady state under sinusoidal excitation, these elements always and under all circumstances produce centrally symmetric PHLs of type II. This remarkable regularity is stronger than the previously published rules for the so-called native behavior of the element. Native behavior is the prevailing behavior, to which exceptions are possible. The above observation, represented by Theorem 2, is unique in the sense that it holds without any exceptions.

The phenomenon of a frequency-doubled parameter occurs when the signals of the state variable ***x*** and excitation *u* are processed by the nonlinearity of the element parameter *P*(***x***,*u*). The phenomenon can be caused by a favorable type of this nonlinearity and can occur even when the frequency of the state variable is not doubled. However, this requires that the nonlinearity of the parameter *P* does not generate a harmonic component whose frequency is equal to the excitation frequency. Another example, according to Theorem 6, is a parameter that is an even function of the excitation *u*. This specific condition is automatically satisfied for generic elements since their parameters do not depend on the excitation at all. On the other hand, doubling the frequency of the state variable does not, by itself, induce the effect.

For many practical applications of extended HOEs, there is an analytical description of the relevant theoretical concepts or physical principles that determine the behavior of the element. Examples include the Corsage memristors [76] used in neuromorphic applications or various types of resistive switching [86]. In such cases, the defining relations (1) and (3) of the element are available, and the test sequences (6) and (7) can be generated, respectively. An investigation into the sequence then leads to the determination of the type of PHL, including the order of arm touching. Examples are given in Section 5.3 and Section 6.3.

## Data Availability

Not applicable.
